# What limits limits?

**DOI:** 10.1093/nsr/nwaa210

**Published:** 2020-08-28

**Authors:** Yong-Chun Liu, Kun Huang, Yun-Feng Xiao, Lan Yang, Cheng-Wei Qiu

**Affiliations:** State Key Laboratory of Low-Dimensional Quantum Physics, Department of Physics, Frontier Science Center for Quantum Information, Tsinghua University, China; Department of Optics and Optical Engineering, University of Science and Technology of China, China; State Key Laboratory for Mesoscopic Physics and Frontiers Science Center for Nano-optoelectronics, School of Physics, Peking University, China; Department of Electrical and Systems Engineering, Washington University, USA; Department of Electrical and Computer Engineering, National University of Singapore, Singapore

There are fundamental rules and principles setting the limits of physical systems. It triggers an interesting thought—can we break the limits under specific circumstances? A realistic system can only provide limited functionalities because its performance is physically constrained by some fundamental principles. ‘Breaking the limit’, which usually implies that the capability of a system could be enhanced significantly, is intriguing in many research areas such as high-precision metrology, imaging and nanophotonics.

The claim of ‘breaking the limit’ can be generally divided into three categories. Firstly, the broken limit (such as standard quantum limit) is just a technical limit, but not a fundamental limit governed by basic physical principles (Fig. 1a). Secondly, some limits (e.g. diffraction limits in optical focusing and imaging) are broken at the cost of sacrificing other performances of a system, as depicted in Fig. 1b. Thirdly, most limits are generally derived with some prerequisites or assumptions. Once the working scenarios are changed or go beyond the prerequisite condition, the previous limit becomes invalid so that it can be broken (see Fig. 1c). It would lead to some misunderstandings if the limits, such as the Heisenberg limit, time-bandwidth limit and efficiency limit, of metasurfaces were not elaborated precisely.

**Figure 1.**
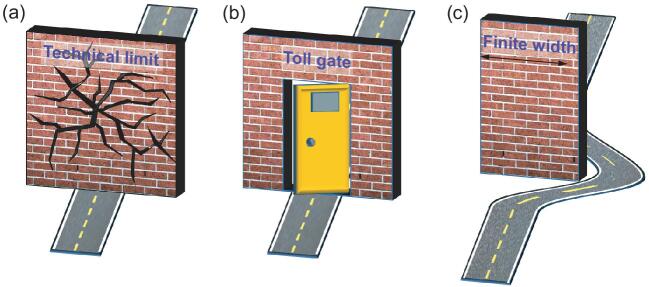
A summary of the three categories of ‘breaking the limits’. (a) The broken limit is a technical limit or pseudo-limit. (b) The limit is broken at the cost of sacrificed performances. (c) The limit is valid under some special conditions. Once the situation is changed, the limit in this case could be automatically broken.

## STANDARD QUANTUM LIMIT

The term ‘standard quantum limit’ was first used to characterize the quantum noise in gravitational wave detectors.

Since then, it has been commonly employed in measurement techniques using photons or atoms [1]. This limit originates from the discrete nature of the measurement results. In quantum optics, the standard quantum limit is related to the shot noise of photons since the light is composed of individual photons. For a classical laser with *N* photons, the measurement outcome has a fluctuation scaling as *N*^1/2^ on average, which is equivalent to the statistic variation of *N* times independent measurements. In a two-level (pseudo-spin) atomic system, the standard quantum limit is related to the spin projection noise, where the projection measurement of *N* individual atoms also leads to a fluctuation of *N*^1/2^. In both cases, the measurement precision scales as *N*^1/2^/*N *=* N^−^*^1/2^. More generally, the precision of measurement scaling with *N^−^*^1/2^ is considered the standard quantum limit.

**Figure 2.**
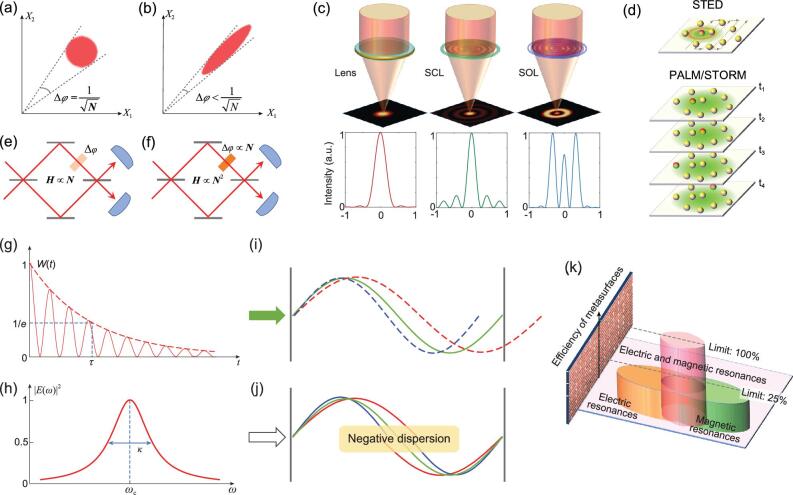
(a, b) Phase-space pictures of the variance of photons for a coherent state (a) and a squeezed state (b); a coherent state corresponds to the standard quantum limit, while the squeezed state breaks the standard quantum limit. (c) Optical focusing beyond the Rayleigh diffraction limit. A spherical lens could focus light into an airy spot. With careful designing, a super-critical lens (SCL) could achieve a smaller spot but with a visible side lobe. The super-oscillatory lens (SOL) can push the central spot down to the deep-subwavelength scale; however, the side lobe would also increase dramatically, making it difficult to use in the industry. (d) Illustration of the basic principle of super-resolution microscopies such as stimulated emission depletion (STED) microscopy, photoactivated localization microscopy (PALM) and stochastic optical reconstruction microscopy (STORM). The red (yellow) spheres denote fluorescent (non-fluorescent) objects. The area of illumination is marked in green. (e) Linear Mach-Zehnder interferometer with the Hamiltonian scales as a linear function of photon number *N*. The two optical paths have a phase difference Δ*φ*, which does not depend on *N*. (f) Non-linear Mach-Zehnder interferometer with the Hamiltonian scales as a quadratic function of photon number *N*. The two optical paths have a phase difference Δ*φ* proportional to *N*. (g) Energy decay in the resonator in the time domain, where *τ* is the energy storage time. (h) The spectrum of a resonance mode in the frequency domain, where *ω*_c_ is the resonance frequency and *κ* is the resonance bandwidth. (i) Conventional optical resonator with a discrete resonance frequency that satisfies the resonant condition. (j) White-light optical resonator, which contains a negative dispersion medium inside the resonator. Light with different frequencies can be on resonance due to the compensation of the phase delay, leading to more condensed resonances in the spectrum. (k) Efficiency limit of optical metasurfaces. The limit for metasurfaces supporting either electric or magnetic resonance is 25%, while the efficiency of high aspect-ratio dielectric and multilayer metasurfaces could approach 100% in theory.

However, the standard quantum limit is not a fundamental limit. It can be viewed as a technical limit of a system measured in the coherent state. After introducing quantum manipulations, the standard quantum limit can be exceeded. For example, when the photons (or atoms) are in a squeezed state, the variance of the measurement results can be smaller in a certain direction in the quadrate phase space (Fig. 2a and b). In this case, the photons (or atoms) have quantum correlations and behave cooperatively, and they cannot be viewed individually. Another example is the NOON state, which is a superposition state of the form (| N,0〉+| 0, N〉)/2^1/2^. The output state becomes (| N,0〉 e*^iN^^φ^*+| 0, N〉)/2^1/2^, where the accumulated phase is multiplied by *N* compared with the general coherent state case. It then enables the precision of measurement scaling as 1/*N*, which is further limited by the Heisenberg limit.

## DIFFRACTION LIMIT

In a lens-based microscopic system, the resolving power in optical focusing and imaging is limited to ∼0.5*λ*/*NA*, where *λ* is the wavelength of light and *NA* is the numerical aperture of the lens. Focusing light into a hot spot is important in imaging as well as in nanofabrication. Recent progress has suggested that the hot spot could be reduced down to the

deep-subwavelength scale by using the super-critical and super-oscillatory lenses [2], as shown in Fig. 2c. For the super-critical lens (SCL), the focal spot approaches the super-oscillation criterion of 0.38*λ*/*NA*. Meanwhile, the sidelobe of the SCL spot is still below 16.2% of its main lobe, which is acceptable in most applications. However, for the super-oscillation lens (SOL), the much stronger sidelobe is a serious barrier to industrial use. Consequently, the benefits of high-resolution central main lobes are compromised or even voided. Due

to energy conservation, the reduction of the central main lobe size in an *NA*-specific lens will reduce the energy confined within the main lobe so that the energy is redistributed to the side lobe. Therefore, the criterion of 0.38*λ*/*NA* becomes the ultimate limit of optical focusing from the viewpoint of engineering and applications.

Similarly, the imaging resolution of a traditional microscope is usually limited to ∼200 nm at visible wavelengths due to the diffraction of light. Since the focal spot achieved by the interference of multiple beams suffers from the impassable limit, the super-resolution techniques turn to utilizing other properties of light (e.g. spectrum) in combination with pre-processed specimens. Super-resolution microscopy techniques, such as stimulated emission depletion (STED), photoactivated localization microscopy (PALM) and stochastic optical reconstruction microscopy (STORM) [3], selectively activate one fluorescent object within the diffraction zone of 0.5*λ*/*NA* and read them out sequentially (STED) or randomly (PALM/STORM) (Fig. 2d). Then, every object can be precisely localized by calculating the intensity centroid of its recorded fluorescence pattern. Although the imaging resolution could be improved to be tens of nanometers, the time to map a full picture with fine details increases significantly due to the scanning or time-sequential readout mode, as compared with the one-shot direct imaging by an objective lens. Since the pre-labelling operation of targeted objects is required, these techniques are usually valid for the fluorescent samples and particularly popular in life sciences. In addition, although a superlens could also reconstruct the deep-subwavelength details of objects by using evanescent waves [2], the working distance is limited within one wavelength. Hence, the super-resolution imaging is achieved at the cost of the time-consuming image-taking process, narrowly ranged samples or limited working distance.

## HEISENBERG LIMIT

The term ‘Heisenberg limit’ was first introduced to describe the limit resulting from the Heisenberg uncertainty relation [4]. Since the Heisenberg uncertainty relation is a fundamental principle of quantum mechanics, Heisenberg limit should be an ultimate limit in measurement precision, which scales as 1/*N*, with *N* being the particle number. However, this result is based on the linear interferometer model, where the parameters to be measured are encoded in the linear evolution Hamiltonian, such as optical Mach-Zehnder interferometer and atomic Ramsey interferometer. When the parameters are encoded in the non-linear interaction or many-body interaction processes, the Heisenberg uncertainty relation does not lead to 1/*N* scaling in measurement precision. Instead, the limit scales as 1/*N^k^* for *k*-body interaction systems (*k* > 1) [5]. Typical examples include the measurement of the interaction strength in Bose-Einstein condensates and the Kerr non-linear coefficient of optical materials. Taking the latter as an example, the phase difference accumulation Δ*φ* is proportional to the non-linear refractive index Δ*n*, which, in turn, is proportional to the light intensity and thus the photon number *N* (Fig. 2e). Therefore, compared with the linear interferometer, here the phase difference has another *N*-fold enhancement, which leads to *N*-fold reduction in the measurement precision. Such ‘super-Heisenberg limit’ breaks the 1/*N* scaling law, but does not violate the Heisenberg uncertainty relation.

## TIME-BANDWIDTH LIMIT

Time-bandwidth limit inherently exists in resonant systems, ranging from optical cavities to mechanical systems and LC circuits. Such a system is typically described by two parameters, resonance frequency *ω*_c_ and quality factor *Q*. The latter is defined as the ratio of the stored energy to the decayed energy in each oscillation cycle. Typically, the energy decays exponentially, i.e. *W*(*t*) = *W*(0)*e*^−^*^κ^^t^*, where *κ* is the energy decay rate. The energy storage time is proportional to the *Q,* i.e. *τ =* 1/*κ *= *Q*/*ω*_c_ (Fig. 2g). In the frequency domain, the energy spectrum of the resonance mode has a Lorentzian line shape, with the central frequency *ω*_c_ and linewidth (full width at half maximum, FWHM) equal to the energy decay rate *κ*, derived from the Fourier transform of the energy spectrum from the time domain to the frequency domain (Fig. 2h). Since *τ *and* κ* have the relation *τ κ *= 1, the product of the energy storage time and the resonance bandwidth is always a constant, leading to the so-called ‘time-bandwidth limit’.

One approach to effectively break the time-bandwidth limit is to use a ‘white-light cavity’ [6]. By designing the dispersion characteristics of the cavity material, for example, introducing negative dispersion, the resonance frequency range can be effectively extended. Normally, the phase delay as a function of wave frequency is given by *φ *= *nωL*/*c*, where *n* is the refractive index of the material, *L* is the propagation length and *c* is the speed of light in a vacuum. A standing-wave optical resonator with length *L* requires the resonance condition *nωL*/*c = 2q*π, resulting in the discrete resonance frequencies *ω*_c _= 2*q*π*c*/*nL*, where *q* is a positive integer (Fig. 2i). When a negative dispersion medium is used to compensate for the ordinary phase delay, the resonance condition becomes *nωL*/*c +φ*_dis_(*ω*)*  = k*π. In this case, the system can support many closely spaced resonances to effectively give a broadband appearance (Fig. 2j). While each individual resonance still respects the time-bandwidth limit, the whole system appears to surpass the limit as the effective bandwidth becomes larger. In other words, the time-bandwidth limit is not broken in any sense.

Recently, Tsakmakidis *et al*. reported a method to break the time-bandwidth limit by using a non-reciprocal resonator with unequal in-coupling and out-coupling rates [7]. However, further studies show that non-reciprocity does not lead to the breaking of the time-bandwidth limit [8]. On the other hand, if a system is varied in time, the time-bandwidth limit for a single resonance can be broken since the resonant frequencies are not constants in a time-varying system [8].

## EFFICIENCY LIMIT

As a 2D version of 3D diffractive optical elements, ultrathin metasurfaces are composed of spatially varied nanostructures that could realize the conversion from the incident polarization to its orthogonal polarization. The conversion efficiency plays a crucial role in real applications of metasurfaces. If the subwavelength structures could only support either electric or magnetic resonances, the theoretical limit of conversion efficiency is 25% [9], which is valid for metal-based single-layer metasurfaces. In fact, when both electric and magnetic resonances are excited simultaneously within the subwavelength structures with high-index materials, high aspect ratio or multilayer configurations (Fig. 2k), the largest efficiency is 100% [10], which becomes the efficiency limit of metasurfaces.

In summary, limit-breaking discoveries seem to imply revolutionary improvements, but we need to carefully check what kind of limit is broken and at what price. By thoroughly analyzing different scenarios, we show that ‘breaking the limit’ has different meanings, which can be categorized into three typical cases. Firstly, the limit is a technical limit but not a fundamental limit. Secondly, the limit is broken by paying the price of sacrificing other performances. Thirdly, the limit seems to break by changing the prerequisites, while the original context of said limit has been modified. With the rapid development of science and technology, we could expect faster progress in breaking more limits; however, we need to be cautious of what actually limits those limits.
